# Correction: Modeling circuit mechanisms of opposing cortical responses to visual flow perturbations

**DOI:** 10.1371/journal.pcbi.1014136

**Published:** 2026-03-30

**Authors:** J. Galván Fraile, Franz Scherr, José J. Ramasco, Anton Arkhipov, Wolfgang Maass, Claudio R. Mirasso

There are errors in the Funding statement. The correct Funding statement is as follows: JGF, and CRM acknowledge support from the Spanish Ministerio de Ciencia, Innovación y Universidades, project PID2021–128158NB-C22 funded by the MICIU/AEI/10.13039/501100011033. JGF, JJR and CRM acknowledge support from the Spanish Ministerio de Ciencia, Innovación y Universidades, project María de Maeztu CEX2021–001164-M funded by the MICIU/AEI/10.13039/501100011033. The contribution of WM and FS was partially supported by the Human Brain Project (Grant Agreement number 785907) of the European Union. The work by AA was supported by the National Institute of Biomedical Imaging and Bioengineering of the National Institutes of Health under Award Number R01EB029813 and the National Institute of Neurological Disorders and Stroke of the National Institutes of Health under Award Numbers R01NS122742 and U24NS124001. The funders did not play any role in the study design, data collection and analysis, decision to publish, or preparation of the manuscript.
