# Supplementary figures and images for: Modeling circuit mechanisms of opposing cortical responses to visual flow perturbations
Source: PLoS Comput Biol. 2024 Mar 7;20(3):e1011921. doi: 10.1371/journal.pcbi.1011921 (PMC10950248; doi:10.1371/journal.pcbi.1011921)

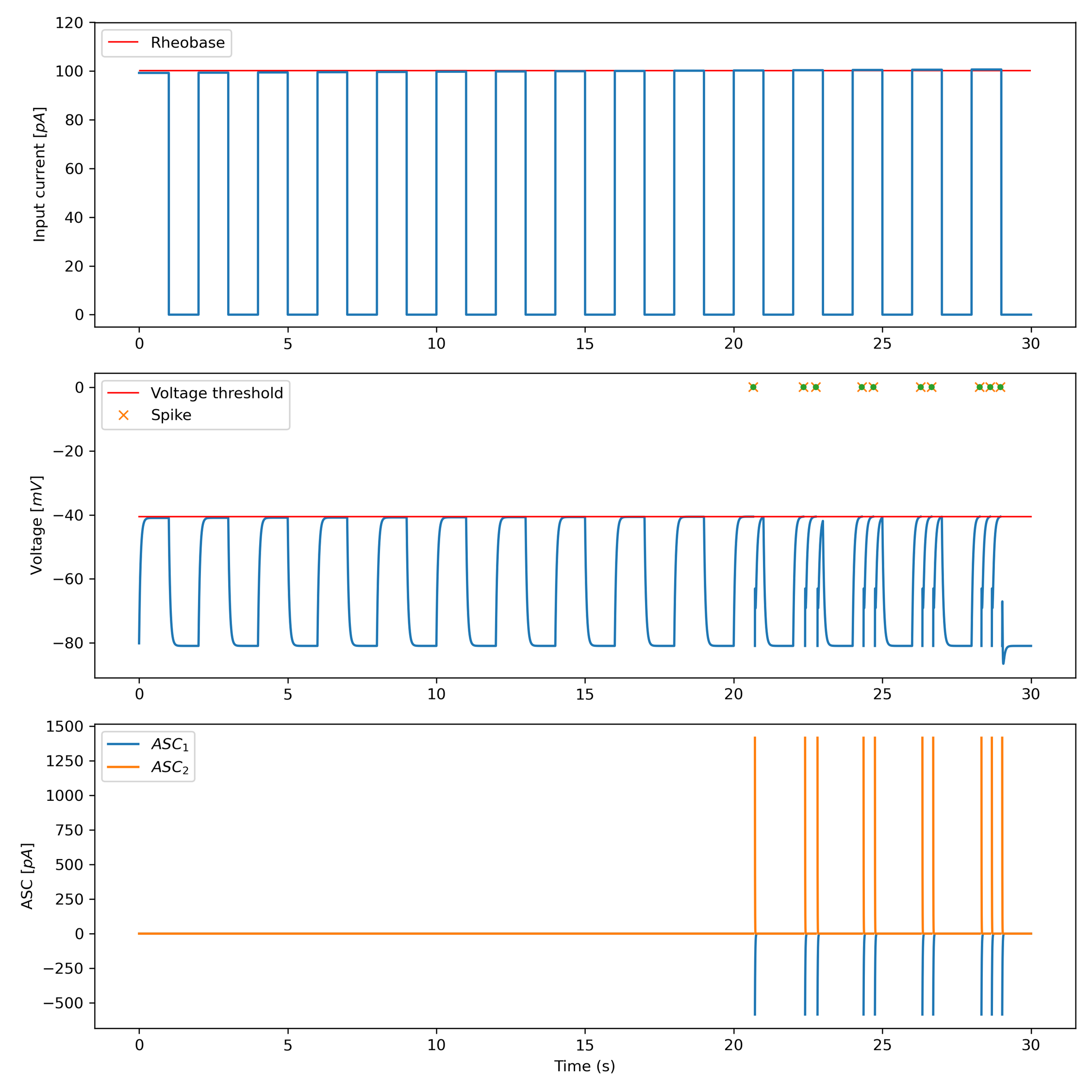

Supplement: S1 Fig — Top: Injected current into the cell soma consisting of increasingly larger current steps interleaved by resting periods. The red line represents the identified rheobase. Middle: Cell model membrane voltage as a response to the injected current. The red line represents the voltage threshold in the model. Bottom: After-spike currents of the model. Sharp vertical lines indicate the presence of a spike. (TIF) [file pcbi.1011921.s001.tif]

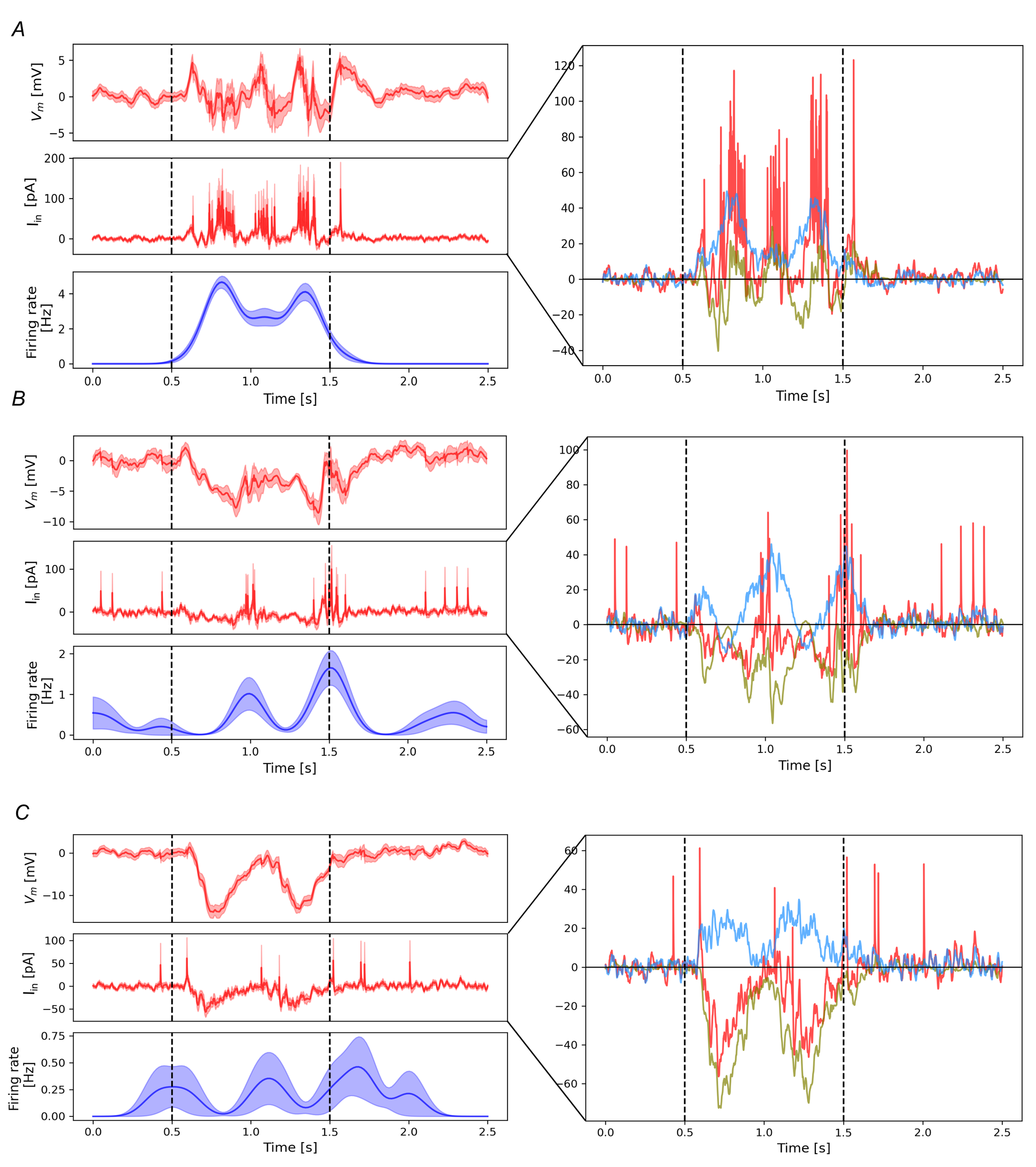

Supplement: S2 Fig — (A) Left: Neuron’s membrane voltage response (top), input current response (middle), and firing rate (bottom) for a sample dVf neuron. The mean input current during visual flow (blue horizontal line) and the classification thresholds (black dots) are shown. Right: Responses of the different PSC sources for a sample dVf neuron, including total input current (red), recurrent current (olive), and LGN current (blue). Vertical dashed lines mark the visual flow period. (B) As in (A), but for a sample unclassified neuron. (C) As in (A), but for a sample hVf neuron. (TIF) [file pcbi.1011921.s002.tif]

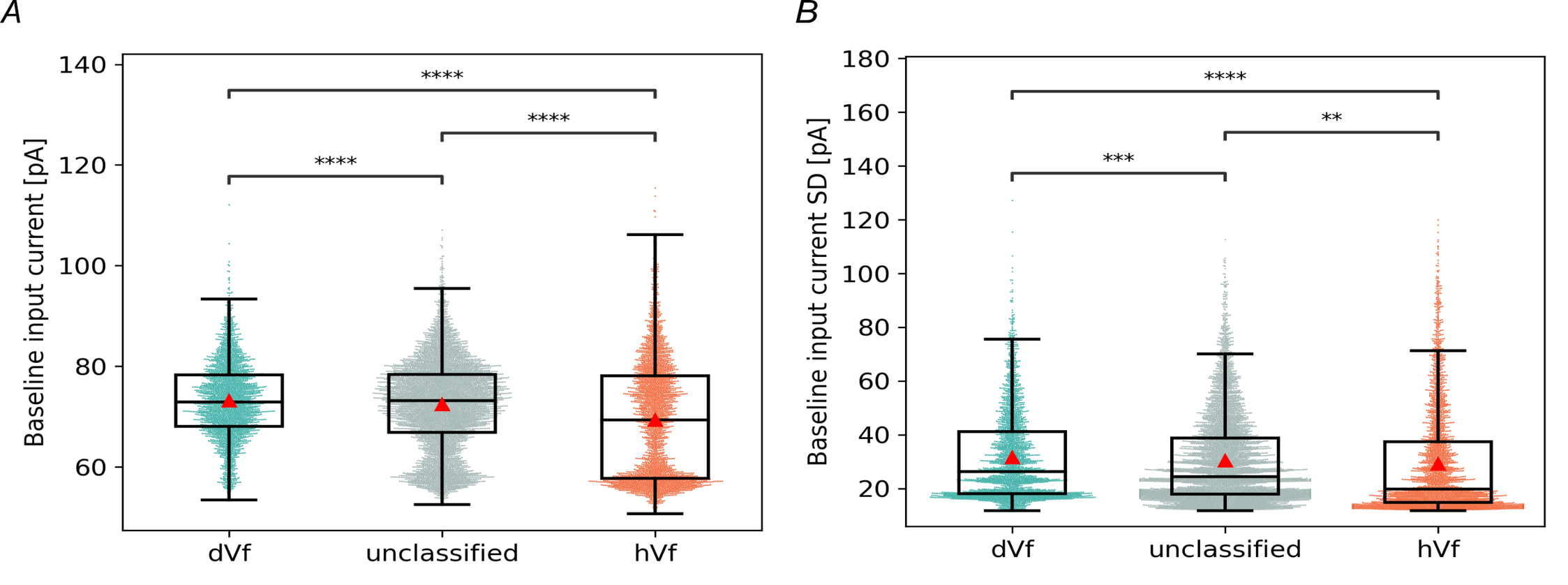

Supplement: S3 Fig — (A) Distribution of baseline input current mean for excitatory L2/3 classes (ANOVA: F = 162.8, p = 1.5 × 10^−70^, n = 12689; dVf vs hVf: t = 15.72, p = 1.5 × 10^−54^, n = 6311; dVf vs unc: t = 4.18, p = 2.9 × 10^−5^, n = 9326; hVf vs unc: t = 13.6, p = 2.8 × 10^−41^, n = 9741, Welch’s t test). (B) Distribution of baseline input current standard deviation (SD) for excitatory L2/3 classes (ANOVA: F = 14.96, p = 3.2 × 10^−7^, n = 12689; dVf vs hVf: t = 5.24, p = 1.7 × 10^−7^, n = 6311; dVf vs unc: t = 3.58, p = 3.5 × 10^−4^, n = 9326; hVf vs unc: t = 2.72, p = 0.0065, n = 9741, Welch’s t-test). Red triangles indicate the class mean. (TIF) [file pcbi.1011921.s003.tif]

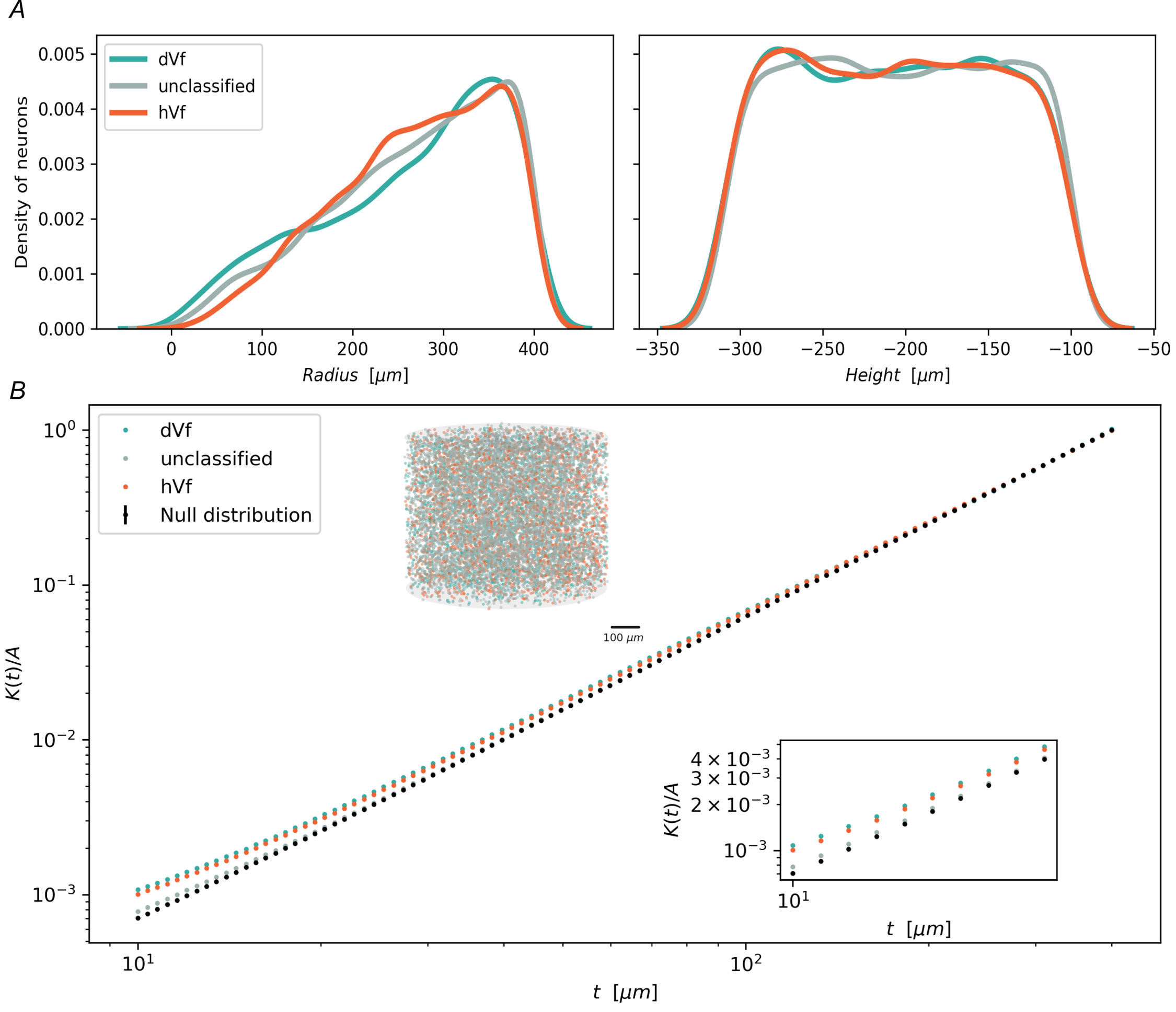

Supplement: S4 Fig — (A) The density distribution of the distance to the center of the column, which we refer to as radius, (left), and the depth within the layer (right) is depicted for dVf, hVf, and unclassified neurons. (B) Ripley’s K divided by the layer 2/3 area as a function of the search radius t. The colors of the curves and dots represent the results for dVf (turquoise), hVf (orange), and unclassified (gray) neurons. The black points represent the outcome of a random null model of 10,000 neurons averaged over 100 realizations. Also, the excitatory L2/3 classes are represented in the V1 cylinder. (TIF) [file pcbi.1011921.s004.tif]

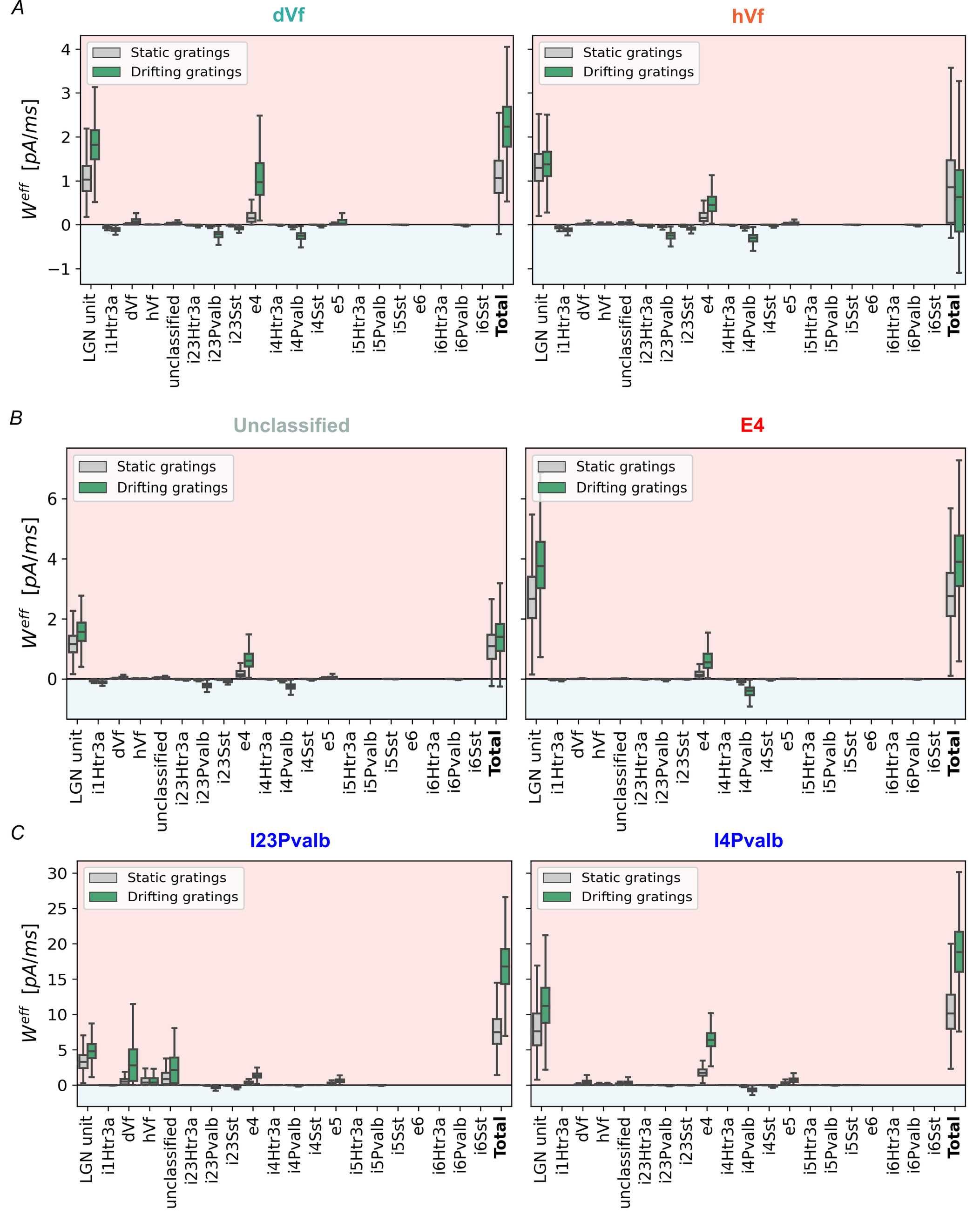

Supplement: S5 Fig — Effective synaptic weight for various neuron types: (A) dVf and hVf neurons; (B) L2/3 unclassified and L4 excitatory neurons; and (C) L2/3 and L4 inhibitory Pvalb neurons. The bars represent the effective weight 500ms before (orange) and 500ms after (purple) the onset of visual flow. (TIF) [file pcbi.1011921.s005.tif]

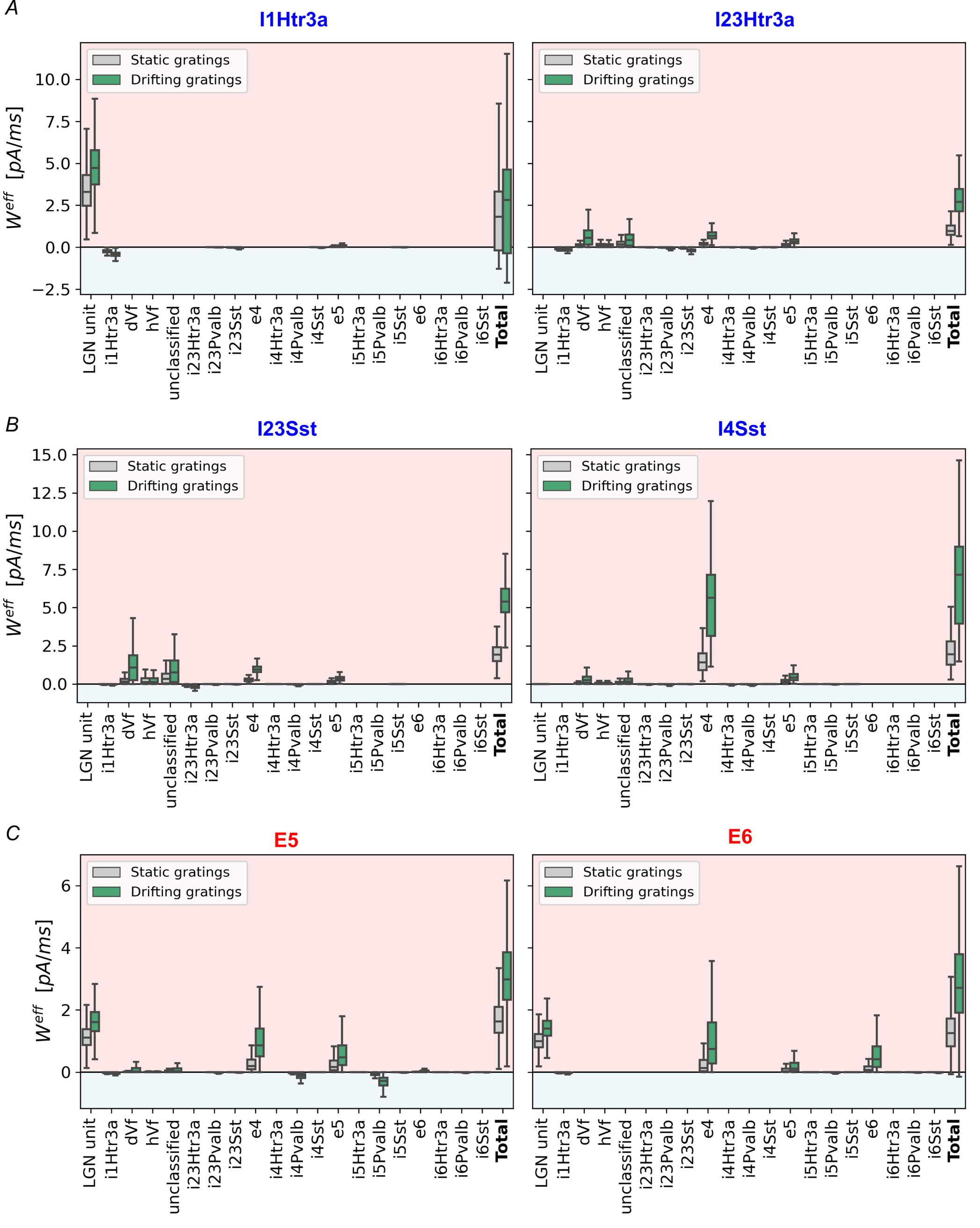

Supplement: S6 Fig — Effective synaptic weight for various neuron types: (A) L1 and L2/3 inhibitory Htr3a neurons; (B) L2/3 and L4 inhibitory Sst neurons; and (C) excitatory L5 and L6 neurons. The bars represent the effective weight 500ms before (orange) and 500ms after (purple) the onset of visual flow. (TIF) [file pcbi.1011921.s006.tif]

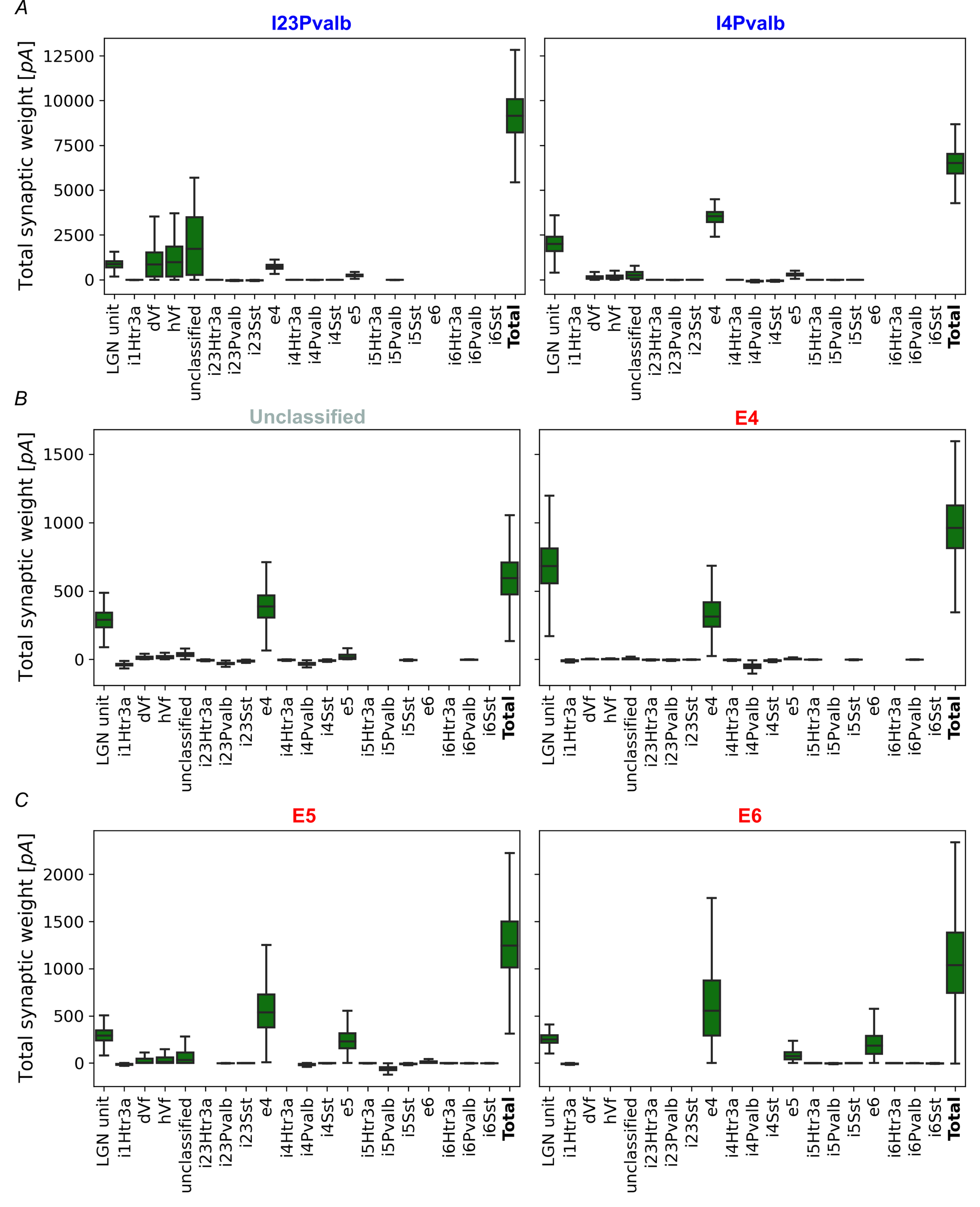

Supplement: S7 Fig — Total synaptic weight of several presynaptic populations for: (A) L2/3 and L4 inhibitory Parvalbumin neurons; (B) L2/3 unclassified and excitatory L4 neurons; and (C) excitatory L5 and L6 neurons. (TIF) [file pcbi.1011921.s007.tif]

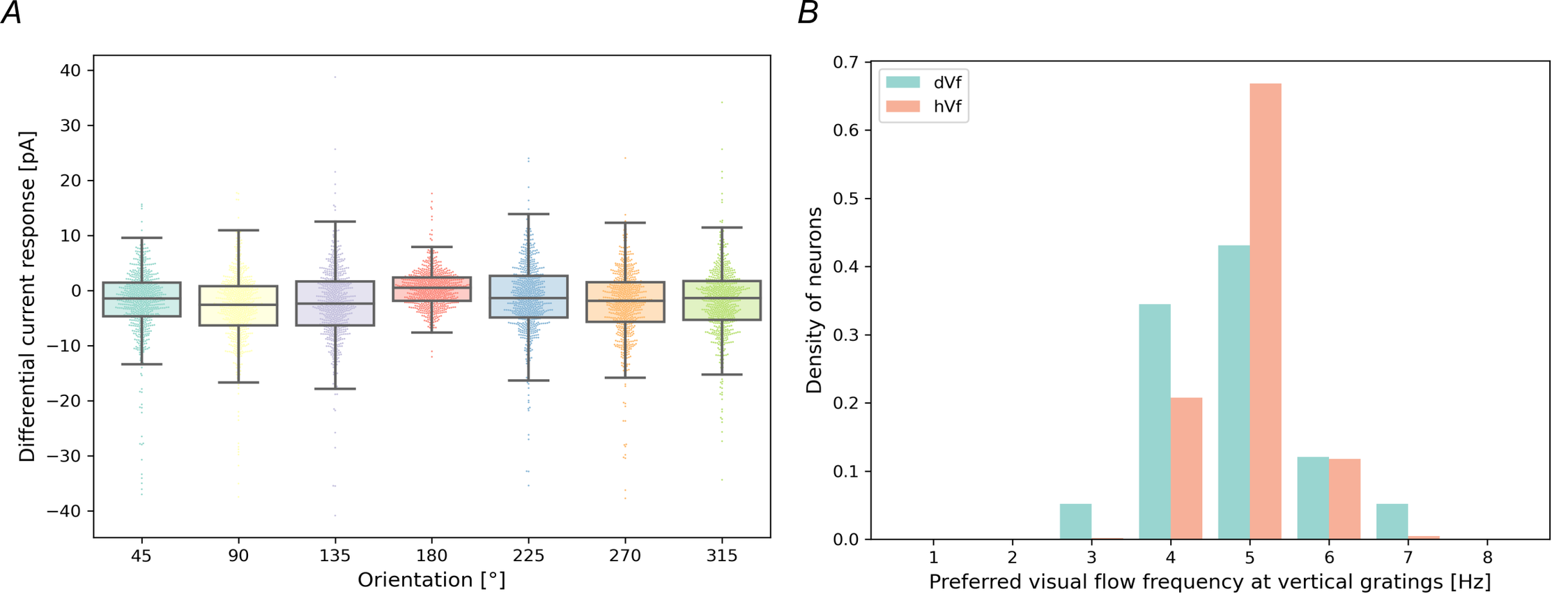

Supplement: S8 Fig — (A) Input current responses in various drift directions normalized to the input current responses to horizontal drift (0° direction). (B) Distribution of preferred visual flow frequency of dVf (turquoise) and hVf (orange) neurons, and horizontal drift vertical gratings. (TIF) [file pcbi.1011921.s008.tif]

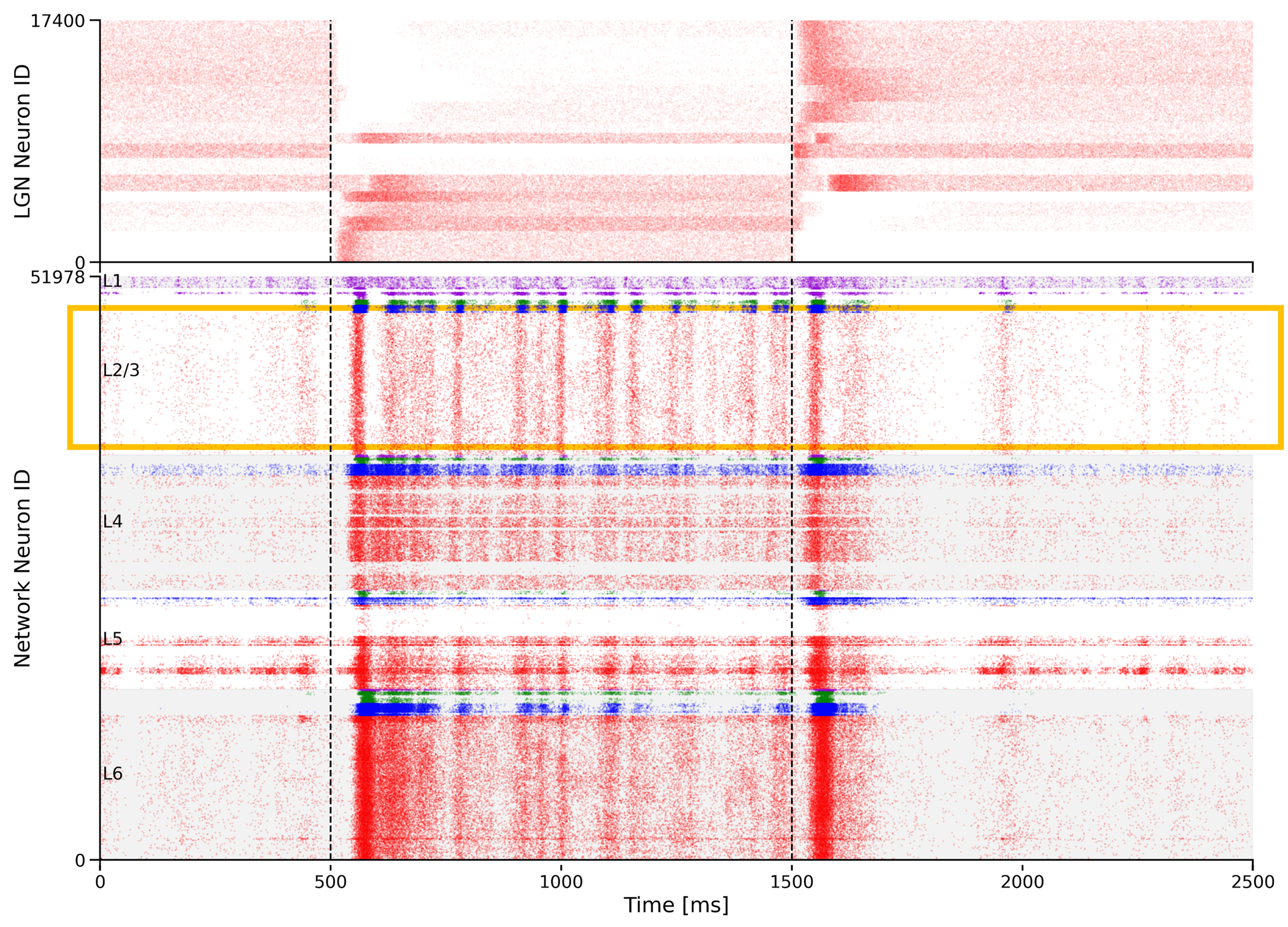

Supplement: S9 Fig — Top: Raster plot of the spike response of LGN units to full-field flash. Bottom: Laminar raster plot of the spike response of V1 neurons to full-field flash. The colors of the spikes represent the different populations of neurons, following the same palette as in Fig 1. Vertical dashed lines indicate the period of full-field flash. (TIF) [file pcbi.1011921.s009.tif]
