# Supplementary material for: Modeling circuit mechanisms of opposing cortical responses to visual flow perturbations
Source: PLoS Comput Biol. 2024 Mar 7;20(3):e1011921. doi: 10.1371/journal.pcbi.1011921 (PMC10950248; doi:10.1371/journal.pcbi.1011921)
Supplement: S1 Table — Mean values for the different PSC sources during the static gratings (baseline) and drifting gratings (visual flow) periods. In some populations, a classification of the belonging neuron’s response to the drifting gratings was made (see Methods for details). The SEM on the sample population and realizations is taken as the error. The total current considers the contributions of the recurrent, bottom-up, ASC and BKG currents. (PDF) [file pcbi.1011921.s010.pdf]

S1 Table. Input currents for different neuron populations.

			Baseline currents [pA]			Visual flow currents [pA]		
Population	Class	Abundance	Recurrent	BU	**Total**	Recurrent	BU	**Total**
E2/3	dVf	23.2%	0.32±0.06	14.8±0.1	**73.2±0.1**	2.5±0.2	25.3±0.2	**84.4±0.2**
	hVf	26.5%	-0.34±0.06	11.6±0.2	**69.3±0.2**	-9.8±0.1	12.2±0.2	**60.9±0.2**
	unc	50.3%	-0.10±0.04	14.4±0.1	**72.4±0.1**	-5.1±0.1	19.3±0.1	**72.2±0.1**
I2/3 Pvalb	dVf	100%	35.9±0.5	20.4±0.5	**72.3±0.8**	91.0±0.7	29.1±0.7	**133±1**
I2/3 Htr3a	dVf	70%	6.8±0.1	0	**25.1±0.2**	19.2±0.2	0	**35.1±0.3**
	unc	30%	6.3±0.1	0	**28.4±0.4**	13.9±0.2	0	**35.9±0.5**
I2/3 Sst	dVf	100%	15.0±0.2	0	**50.2±0.5**	39.6±0.4	0	**77.2±0.6**
E4	dVf	52.4%	0.2±0.0	36.9±0.2	**35.7±0.2**	-1.1±0.1	60.5±0.2	**51.5±0.2**
	hVf	10.9%	1.6±0.1	52.1±0.5	**47.8±0.4**	-5.8±0.2	50.4±0.5	**36.9±0.4**
	unc	36.7%	0.3±0.0	43.3±0.2	**41.5±0.2**	-4.1±0.1	51.5±0.3	**42.9±0.2**
I4 Pvalb	dVf	96.1%	17.9±0.2	58.6±0.8	**72.9±0.7**	51.3±0.4	83.6±0.9	**118.5±0.7**
	unc	3.9%	23±1	101±4	**110±4**	49±2	89±4	**117±3**
E5/6	dVf	62.7%	9.0±0.1	13.4±0.1	**95.7±0.2**	34.4±0.2	19.8±0.1	**115.6±0.2**
	hVf	0.3%	15±3	21.2±0.9	**80±3**	8±3	21.8±0.7	**71±3**
	unc	37.0%	7.2±0.1	14.1±0.1	**93.2±0.2**	13.6±0.1	18.0±0.1	**99.6±0.2**

Mean values for the different PSC sources during the static gratings (baseline) and drifting gratings (visual flow) periods. In some populations, a classification of the belonging neuron's response to the drifting gratings was made (see Methods for details). The SEM on the sample population and realizations is taken as the error. The ASC and BKG inputs are not shown but do contribute to the total current.
