# Supplementary material for: Modeling circuit mechanisms of opposing cortical responses to visual flow perturbations
Source: PLoS Comput Biol. 2024 Mar 7;20(3):e1011921. doi: 10.1371/journal.pcbi.1011921 (PMC10950248; doi:10.1371/journal.pcbi.1011921)
Supplement: S2 Table — Connections with other V1 neurons (recurrent), thalamus (LGN), and noisy background sources (BKG) are considered. The SEM for each variable is taken as the error. (PDF) [file pcbi.1011921.s011.pdf]

S2 Table. In/Out degrees for excitatory L2/3 classes.

Class	Out-degree (k_{out})	In-degree (k_{in})			
		Recurrent	LGN	BKG	**Total**
dVf	337.7±0.3	469.3±0.4	16.5±0.1	1.0±0.0	**486.8±0.4**
hVf	338.1±0.3	471.9±0.4	8.7±0.1	1.0±0.0	**481.6±0.4**
unc	337.5±0.2	469.3±0.3	13.2±0.1	1.0±0.0	**483.5±0.3**

Connections with other V1 neurons (recurrent), thalamus (LGN), and noisy background sources (BKG) are considered. The SEM for each variable is taken as the error.
