# Supplementary material for: Modeling circuit mechanisms of opposing cortical responses to visual flow perturbations
Source: PLoS Comput Biol. 2024 Mar 7;20(3):e1011921. doi: 10.1371/journal.pcbi.1011921 (PMC10950248; doi:10.1371/journal.pcbi.1011921)
Supplement: S3 Table — Weighted in/out-degrees, i.e. the number of incoming/outgoing connections multiplied by the synaptic weight, with other V1 neurons (recurrent), thalamus (LGN), and noisy background sources (BKG) are considered. The SEM for each variable is taken as the error. (PDF) [file pcbi.1011921.s012.pdf]

S3 Table. Weighted in/out degrees for excitatory L2/3 classes.

Class	Weighted out-degree (k_{out})	Weighted in-degree (k_{in})			
		Recurrent	LGN	BKG	**Total**
dVf	1067±3	404±3	297±2	4.0±0.0	**704±3**
hVf	1081±3	302±2	157±3	4.0±0.0	**463±3**
unc	1074±2	353±2	237±2	4.0±0.0	**594±2**

Weighted in/out-degrees, i.e. the number of incoming/outgoing connections multiplied by the synaptic weight, with other V1 neurons (recurrent), thalamus (LGN), and noisy background sources (BKG) are considered. The SEM for each variable is taken as the error.
