# Supplementary material for: Modeling circuit mechanisms of opposing cortical responses to visual flow perturbations
Source: PLoS Comput Biol. 2024 Mar 7;20(3):e1011921. doi: 10.1371/journal.pcbi.1011921 (PMC10950248; doi:10.1371/journal.pcbi.1011921)
Supplement: S4 Table — The table presents the statistical significance testing results, providing insights into the microcircuitry differences between dVf and hVf neurons. Welch’s t-test were performed, with the low p-values attributed to the large number of neurons within both dVf and hVf classes. (PDF) [file pcbi.1011921.s013.pdf]

S4 Table. Statistical significance testing results for the comparison of total presynaptic weight sources between dVf and hVf neurons.

Source Population	Comparison Population	p-value	t-value
i1Htr3a	dVf vs. hVf	1.015×10^{-4}	3.889
dVf	dVf vs. hVf	0.0	45.1
unclassified	dVf vs. hVf	1.011×10^{-15}	-8.047
hVf	dVf vs. hVf	0.0	-40.66
i23Htr3a	dVf vs. hVf	0.07806	1.762
i23Pvalb	dVf vs. hVf	2.681×10^{-52}	15.36
i23Sst	dVf vs. hVf	1.558×10^{-19}	9.071
e4	dVf vs. hVf	5.525×10^{-165}	28.27
i4Htr3a	dVf vs. hVf	0.8174	-0.2309
i4Pvalb	dVf vs. hVf	4.571×10^{-85}	19.85
i4Sst	dVf vs. hVf	4.636×10^{-4}	3.503
e5	dVf vs. hVf	1.211×10^{-8}	5.706
i5Sst	dVf vs. hVf	0.8413	-0.2002
i6Pvalb	dVf vs. hVf	1.811×10^{-4}	3.746

The table presents the statistical significance testing results, providing insights into the microcircuitry differences between dVf and hVf neurons. Welch's t-test were performed, with the low p-values attributed to the large number of neurons within both dVf and hVf classes.
