# Supplementary material for: Modeling circuit mechanisms of opposing cortical responses to visual flow perturbations
Source: PLoS Comput Biol. 2024 Mar 7;20(3):e1011921. doi: 10.1371/journal.pcbi.1011921 (PMC10950248; doi:10.1371/journal.pcbi.1011921)
Supplement: S5 Table — The table presents the statistical significance testing results, providing insights into the microcircuitry differences between dVf and hVf neurons. Welch’s t-test were performed, with the low p-values attributed to the large number of neurons within both dVf and hVf classes. (PDF) [file pcbi.1011921.s014.pdf]

S5 Table. Statistical significance testing results for comparing the number of synapses by source type between dVf and hVf neurons.

Source Population	Comparison Population	p-value	t-value
i1Htr3a	dVf vs. hVf	5.553×10^{-3}	-2.774
dVf	dVf vs. hVf	3.729×10^{-65}	17.26
unclassified	dVf vs. hVf	4.762×10^{-5}	-4.070
hVf	dVf vs. hVf	1.247×10^{-44}	-14.13
i23Htr3a	dVf vs. hVf	0.5868	-0.5435
i23Pvalb	dVf vs. hVf	5.626×10^{-34}	-12.22
i23Sst	dVf vs. hVf	1.883×10^{-13}	-7.373
e4	dVf vs. hVf	1.736×10^{-8}	5.644
i4Htr3a	dVf vs. hVf	0.6457	0.4597
i4Pvalb	dVf vs. hVf	2.805×10^{-51}	-15.20
i4Sst	dVf vs. hVf	0.4414	-0.7698
e5	dVf vs. hVf	0.2394	1.176
i5Sst	dVf vs. hVf	0.1345	1.497
i6Pvalb	dVf vs. hVf	1.039×10^{-2}	-2.563

The table presents the statistical significance testing results, providing insights into the microcircuitry differences between dVf and hVf neurons. Welch's t-test were performed, with the low p-values attributed to the large number of neurons within both dVf and hVf classes.
