# Supplementary material for: Modeling circuit mechanisms of opposing cortical responses to visual flow perturbations
Source: PLoS Comput Biol. 2024 Mar 7;20(3):e1011921. doi: 10.1371/journal.pcbi.1011921 (PMC10950248; doi:10.1371/journal.pcbi.1011921)
Supplement: Attachment — Submitted filename: Reviewers response letter.pdf [file pcbi.1011921.s016.pdf]

Claudio Mirasso
Full Professor

T. +34 971 172783
claudio@ifisc.uib-csic.es
https://ifisc.uib-csic.es/es/claudio/

Dr. Lyle Graham
Section Editor,
PLOS Computational Biology

Dear Dr. Lyle Graham,

We are grateful for the opportunity to revise our manuscript titled "Modeling circuit mechanisms of opposing cortical responses to visual flow perturbations" following the insightful feedback received from the reviewers. Their constructive comments have been invaluable in guiding us to refine and enhance our paper, ensuring that it meets the high standards of your esteemed journal. We have carefully considered each point raised and have made comprehensive revisions to address these concerns. Below, we present our responses to each of the comments, outlining the changes made to our manuscript accordingly.

In response to the **comments from Reviewer #1**, we agree that certain aspects of our manuscript require further clarification and rephrasing to better convey our findings. In this respect, we propose the following changes:

1.1. *Figure 1: The quality of the figure should be improved. Text in A cannot be read.*

We acknowledge the issues regarding the readability of Figure 1A. We have taken steps to enhance the figure's quality. Specifically, the resolution has been increased to 300 dpi, and the font size has been adjusted for better legibility. Additionally, we have revised all figures in the manuscript to ensure they meet the 300-dpi standard. We have also prepared .tif files of these improved figures for final submission, ensuring that they meet the publication's quality requirements.

1.2. *Model is general, is a little bit vague. It seems that it is better to first introduce general model including second part of Eq. 1 and then Eq. 2. The first part of Eq. 1 (ASC) should be defined after Eq 2. Furthermore, ACS should be explained in more details since it is not known for the most people in the field and is not exactly explained.*

We appreciate your feedback on the clarity of the model description. In response, we have restructured the Materials and Methods section for better coherence. The general dynamics of the model are now introduced first, followed by detailed explanations of each component, including the After-Spike Currents (ASC). The revised section provides a clearer, step-by-step breakdown of the model, making it more accessible to readers unfamiliar with ASC and its role in the model.

1.3. *Eq. 3 also looks imprecise. In the equations, t should be the time of the firing of the presynaptic neurons. Therefore, it seems more precise that instead of time t, write it in the form of (t-t_sp) where t_sp is the time of firing of the presynaptic neuron or external input.*

Your observation regarding the precision of Eq. 3 is well-taken. In the revised manuscript, we have modified the equation to reflect the discrete nature of the computational implementation more accurately. This includes explicitly incorporating the timing of presynaptic spikes into the equation, making it more intuitive and practical for implementation purposes.

1.4. *Again, in Eq. 5 I_e(t) is written in another form. I think there is some discrepancy in writing the methods. I understand what the authors meant, but for being comprehensible for general reader and for being reproducible a more coherent and precise format in needed.*

We acknowledge the inconsistency in the presentation of Eq. 5 and its potential for confusion. To rectify this, the Materials and Methods section has been thoroughly revised for consistency and clarity. We have tried to ensure that each variable and equation is presented coherently and unambiguously, facilitating a clearer understanding for the general reader and aiding reproducibility.

1.5. *Fig. 3: In the caption, please note what are different columns in part A.*

We have updated the caption for Figure 3, Part A to clarify the different columns. Please see the revised caption for this clarification.

1.6. *Line 325: “hVf neurons lack direct connections to the LGN” should it be from LGN?*

Right. We have made the correction as suggested:

Line 325: "hVf neurons lack direct connections from the LGN."

1.7. *Line 326: “This discovery aligns with” might be “this observation aligns with”.*

We appreciate your suggestion and have made the suggested change in the manuscript.

1.8. *Lines 332-337: I do not agree that this is a rich-club structure. Indeed, this is more looks like a modular structure. In rich-club structure there is a subset of the nodes in different modules that are more strongly connected to each other. Please write this part with more care.*

We appreciate this comment and agree with your observation that the pattern we described in the connections of excitatory L2/3 neurons is more indicative of a modular structure rather than a rich-club organization. We have carefully reconsidered this aspect of our study and we have reinterpreted our findings to emphasize a modular network structure in the dVf and hVf neurons. We now describe how neurons within the same class exhibit a higher probability and strength of connections, aligning with the characteristics of a modular network. This perspective is also supported by recent studies that highlight the importance of modular organization in cortical networks for specialized processing and functional (Yoshimura et al 2018, Kim et al 2018, Hagihara et al 2021). Furthermore, we have incorporated additional references to strengthen our argument. For instance, the work of Negrón et al. (2023) on segregated networks of pyramidal neurons in the mouse primary visual cortex provides evidence of modular patterns in cortical areas.

. Yoshimura Y, Dantzker JL, Callaway EM. Excitatory cortical neurons form fine-scale functional networks. Nature. 2005;433(7028):868–873.
. Kim, M. H., Znamenskiy, P., Iacaruso, M. F., & Mrsic-Flogel, T. D. (2018). Segregated subnetworks of intracortical projection neurons in primary visual cortex. Neuron, 100(6), 1313-1321.
. Hagihara, K. M., Ishikawa, A. W., Yoshimura, Y., Tagawa, Y., & Ohki, K. (2021). Long-range interhemispheric projection neurons show biased response properties and fine-scale local subnetworks in mouse visual cortex. Cerebral Cortex, 31(2), 1307-1315.
. Negrón, A., Getz, M. P., Handy, G., & Doiron, B. (2023). The mechanics of correlated variability in segregated cortical excitatory subnetworks. bioRxiv, 2023-04.

1.9. *Lines 349-355: I would suggest moving this part to a relevant position in Discussion section.*

Great suggestion! We have incorporated this change by relocating the mentioned content to a more appropriate section in the Discussion.

1.10. *From line 391: appearance of these three subtitles here without any prior explanations is not appropriate. Please write and explanations before them why they are chosen for more detailed analysis.*

We acknowledge your concern and have revised the manuscript accordingly. In the previous paragraph, we have now explicitly mentioned the reason for the inclusion of these three subtitles. The revised text reads as follows:

"Therefore, a comprehensive dynamic exploration of the main sources of recurrent current, namely L4 excitatory neurons, L4 Pvalb interneurons, and L4 Pvalb interneurons, is essential to understand the mechanism leading to the segregation of the dVf and hVf neurons."

We believe this revision clarifies the rationale behind the choice of these subtitles.

We have carefully considered the **feedback provided by reviewer #2** and will address the points mentioned in the following point-by-point response:

2.1. *My main comments are regarding how parts of the manuscript are 'framed', or other language use.*

The stimulus paradigm itself is not really testing predictive coding explicitly – the stimulus is stationary, moves, and then stops moving. There is no explicit prediction here about what the stimulus is likely to do, and indeed behavior-related variables are not included in the model. Instead, the paradigm reflects stimulus protocols previously used in studies of predictive coding (e.g. [12] and [16]) and seeks to understand if and how bidirectional responses can arise in the absence of sensorimotor expectation.

The authors state it well in the discussion (line 515) "This study aimed to explore the emergence of distinct perturbation-responsive V1 neuron classes in the absence of a sensorimotor expectation using the Billeh model, a biologically realistic computational model [32]."

I felt that a statement such as this could come much earlier in the introduction. Based on the current introduction which, I found myself expecting simulations that more explicitly test predictive coding of visual flow (i.e. using sensorimotor expectation). Given that the current study is addressing a specific question in the field, I suggest making this objective clearer at an early point in the manuscript, to set appropriate expectations for the reader.

We appreciate your feedback and have taken it into consideration. We apologize for any confusion caused by the initial introduction. We have now revised the Introduction section to provide a clearer and more concise objective statement:

"The focus of our study aligns with the broader context of predictive coding but focuses on a specific aspect: the emergence of distinct classes of neurons in V1 that are responsive to visual flow perturbations in the absence of sensorimotor expectations."

2.2. *The Line 2: "The brain's ability to perceive and respond to rapidly changing visual stimuli is a fundamental aspect of sensory processing, vital for the survival and adaptive behavior of animals in their natural environments* [1].*"*
The reference here (a motion coherence paradigm in mice with recordings in V1) does not seem appropriate to the statement made.

We agree with the reviewer and have removed the reference mentioned. Instead, we have cited the following references, which provide more relevant support for the statement:

1. Reynolds JH, Chelazzi L. Attentional modulation of visual processing. Annu Rev Neurosci. 2004;27:611–647.
2. De Franceschi G, Vivattanasarn T, Saleem AB, Solomon SG. Vision guides selection of freeze or flight defense strategies in mice. Current biology. 2016;26(16):2150–2154.
3. Hoy JL, Yavorska I, Wehr M, Niell CM. Vision drives accurate approach behavior during prey capture in laboratory mice. Current Biology. 2016;26(22):3046–3052.

These references better support the statement regarding the brain's ability to perceive and respond to rapidly changing visual stimuli.

2.3. *At the time the introduction felt longer than it needed to be, and at times unfocused e.g., Line 8:*
"Understanding the neural basis of motion perception and the response to visual flow perturbations represents a critical goal in sensory neuroscience, as it would shed light on the underlying processes of attentional modulation and decision-making [5–7]."

I don't particularly disagree with the statement, but it doesn't seem overly necessary to this specific study.

We have condensed and refocused the Introduction for conciseness. However, we find this statement appropriate for our introduction since it highlights the importance of understanding the neural basis of visual flow perturbations.

2.4. *Line 12: "The precise timing with which the brain perceives unexpected stimuli."*

To say the 'brain perceives' seems strange. Perhaps 'signals' is more appropriate?

We agree and have revised the text accordingly, replacing 'perceives' with 'signals' on Line 12.

2.5. *Line 41 and elsewhere: "This segregation is indicative of the neurons' response to visual flow perturbations."*

I appreciate 'visual flow perturbations' is language previously used in other studies, but I would find the text clearer if it be more explicit, e.g. 'stimulus movement onset/halt'

We appreciate your feedback and consideration of our manuscript. However, we respectfully disagree with the suggested change to replace "visual flow perturbations" with "stimulus movement onset/halt."

The term "visual flow perturbations" is a commonly accepted and recognized phrase in the field, consistent with the established terminology used in the literature (Muzzu, 2021; Vasilevskaya, 2023).

It accurately conveys the concept of changes in visual input that we are addressing in our study. Utilizing this established terminology enhances clarity for readers familiar with the field and ensures consistency with existing literature.

We believe that using this term is crucial for maintaining clarity and consistency within the field. Therefore, we kindly request that the original wording be retained in our manuscript.

- Muzzu T, Saleem AB. Feature selectivity can explain mismatch signals in mouse visual cortex. Cell Reports. 2021;37(1):109772.
- Vasilevskaya, A., Widmer, F. C., Keller, G. B., & Jordan, R. (2023). Locomotion-induced gain of visual responses cannot explain visuomotor mismatch responses in layer 2/3 of primary visual cortex. Cell Reports, 42(3).

2.6. *Line 318: “cortical microcircuits have shown that PE neurons may require a balance between “*

Would be preferable not to use the acronym PE for ease of reading.

We have removed the PE acronym, as recommended, to improve readability.

2.7. *Line 325: “In fact, many hVf neurons lack direct connections to the LGN, indicated by zero in-degree values (Fig. 4A).”*

It would be useful to briefly define ‘in-degree’ explicitly here.

We appreciate the reviewer's suggestion and have added a brief definition of "in-degree" to improve the clarity of our paper. This definition is now included alongside the definitions of "out-degree" and "weighted in/out degrees" for better accessibility.

2.8. *Line 329: “The analysis of the connections of excitatory L2/3 neurons revealed a pronounced 329 tendency for neurons of the same class to exhibit a higher probability (Fig. 4B) and 330 strength (Fig. 4C) of connections.”*

Is this really a pronounced tendency? The differences in probability of connection seem quite small (Fig. 4B). It would be useful to state the % or fractional difference

(i.e. 10% more likely) and test the statistical significance. The strength of synaptic weights seems more 'pronounced' – it would be nice to have these differences tested statistically.

We acknowledge the reviewer's valuable feedback regarding the analysis of the connections of excitatory L2/3 neurons, specifically the concern about the pronounced tendency and the need for statistical significance testing. We have addressed these concerns as follows:

First, it is important to clarify that while the differences in connection probability might appear small at first glance, they hold significance in the context of neural connectivity. In Figure 4B, we depict the probability for two randomly chosen neurons to be connected. Notably, we found that it is approximately 15% more likely for a randomly selected dVf neuron to connect with another dVf neuron compared to connecting with a hVf neuron. This same pattern holds true for random hVf neurons as well.

To provide a more concrete measure of these differences, we have conducted statistical significance tests (two-sided Welch t-test’s). The results are as follows:

- For dVf source neurons targeting dVf vs. hVf target neurons:
  - t-statistic: 17.4
  - p-value: 2.7 x 10^-66
- For hVf source neurons targeting dVf vs. hVf target neurons:
  - t-statistic: -14.1
  - p-value: 3.3 x 10^-44
- Sample sizes: n=6311, n_{dVf}=2948, n_{hVf}=3363

We understand that the p-values may appear exceedingly small; however, it is important to consider the context. The statistical tests assess the null hypothesis that both connection probabilities are equal, e.g., P (target = dVf | source = dVf) = P (target = hVf | source = dVf). Given the considerable number of neurons in both the dVf and hVf populations, each consisting of approximately 3,000 neurons, it is reasonable to conclude that the observed differences are indeed highly significant.

To enhance clarity, we have also taken the reviewer's suggestion to heart by simplifying the figures. Specifically, we have removed the unclassified neurons from Figure 4B and 4C, focusing the visual representation on the dVf and hVf neurons. This adjustment should enable a more direct and clear comparison between these two neuron populations.

We believe that these modifications and the inclusion of statistical results significantly strengthen our analysis and address the concerns raised by the reviewer.

2.9. Line 332: “This pattern indicates a rich club structure in dVf and hVf neurons”

It would be helpful to define ‘rich club’ structure rather than relying on a later example as a definition.

Upon careful consideration, we have revised the manuscript accordingly. The microcircuit structure observed in dVf and hVf neurons is more accurately described as a modular structure rather than a rich club structure (as suggested by reviewer 1). To enhance clarity, we have now provided a clear definition of 'modular structure' in the text.

2.10. Line 344: “However, dVf 345 neurons possess slightly stronger incoming recurrent synapses, primarily due to 346 contributions from excitatory L4 neurons (Fig. 4E) and increased LGN input.”

What is exactly is meant here? Does it mean bottom-up LGN inputs AND recurrent cortical connections. I was confused by this statement. Perhaps a comma is missing after ‘(Fig 4E)’?

We apologize for any confusion caused by the sentence in question, and we acknowledge that a missing comma after '(Fig. 4E)' may have led to the confusion. To clarify, the statement refers to the two main differences between dVf and hVf neurons regarding their presynaptic weights. These differences are primarily due to contributions from excitatory L4 neurons, as indicated in Figure 4E, and increased LGN input.

2.11. Figure 5: These are interesting findings, but I struggled to parse the figures properly. E.g., Figuring out which boxplots belong to which x-axis label is difficult. The two different colors used are also hard to parse for panels this small. Additionally, the different y-axis scales limited comparisons of all the different cell types. Perhaps the data could be replotted in a clearer way, potentially by removing weights that are not significantly different from 0 across all the panels (which could be included as a supplementary figure instead). It would also be beneficial to show which differences between hvf and dvf neurons are statistically significant.

We really appreciated this comment since it was true that the figure was not easy to be read. For that reason, and along with these suggestions, we performed the following changes in the figure:

- Change the colors of the boxplots. Instead of the original orange and violet palette, we moved to a gray and green palette. We consider that the gray color better captures the quiet of the static grating’s stimulus, while the green color provides more energy to represent the visual flow. We also included shading to represent the regions of positive and negative effective synaptic weights.
- To move the attention to the important presynaptic sources, we removed every presynaptic population except of those providing jointly the 95% of the total effective synaptic weight during the visual flow period.
- We considered to include statistical test to elucidate which presynaptic populations contribution change significantly during the visual flow period. However, changes are notable in the figure and, since there is a large number of neurons in each population, we decided not to include the results of this test (which we performed) since all of them were significant as you can see in the following figure. Instead, we mentioned it on the figure caption: “*Notably, the comparison between static and drifting gratings'*

effective synaptic weights is statistically significant for all presynaptic populations, with p-values of 0.0001 or lower.”

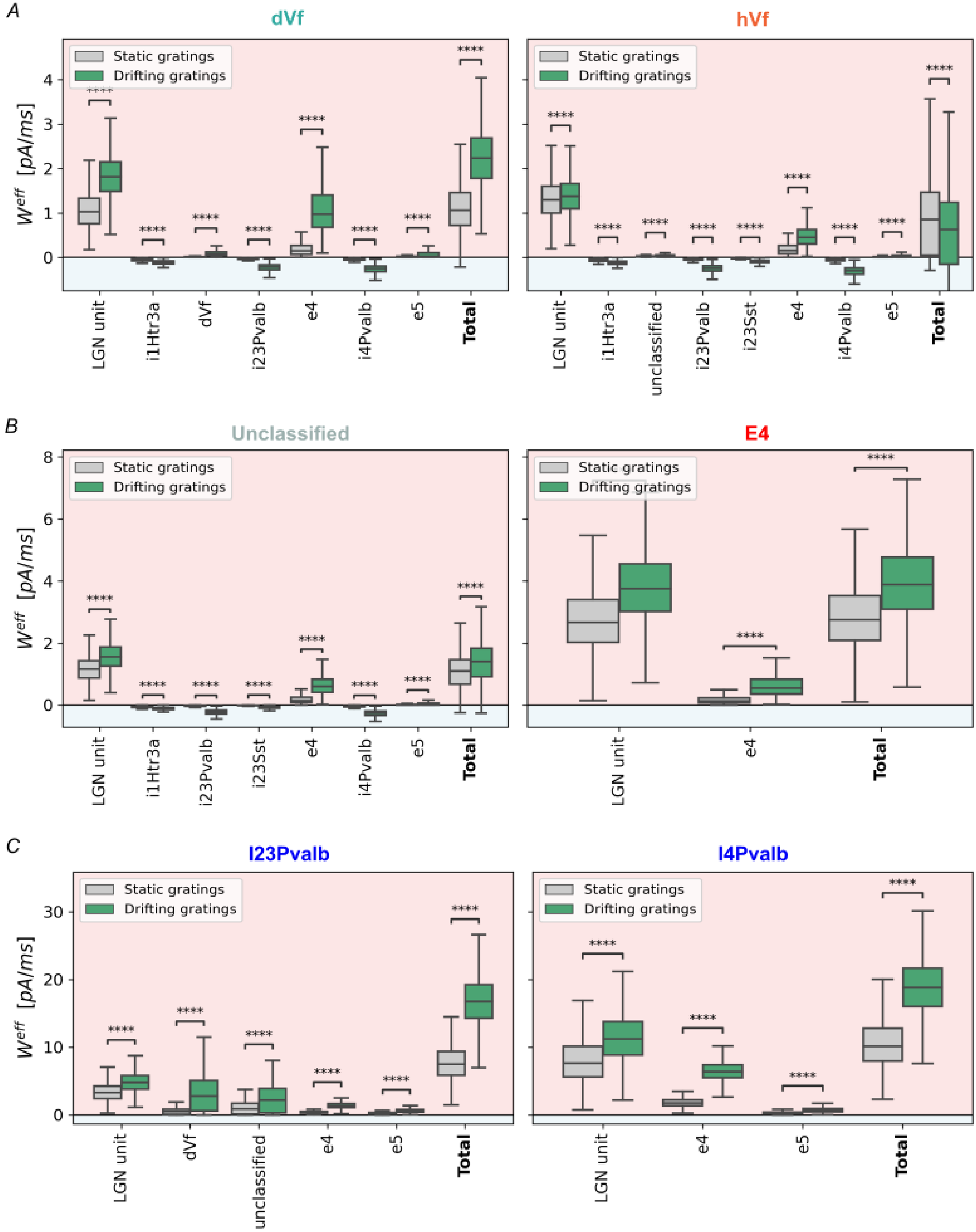

2.12. *Line 431: “To further investigate this, the grating drift speed was increased to 8 Hz, leading to a 431reclassification of excitatory L2/3 neurons based on their response to this new stimulus. 432 Interestingly, we found that neuron assignments to dVf and hVf classes were not 433 significantly affected (Fig. 6B and C), contrary to what was found in mismatch 434 responsive neurons, where their responses scaled linearly with the difference between 435 running speed and visual flow speed [11]. This observation underscores a specific 436 limitation in the Billeh model when processing different temporal dynamics of visual 437 stimuli.”*

Given that no relationship between visual speed and running speed is established in the simulation (indeed, as far as I can tell, behavior is not explicitly modelled in any way), to imply that higher temporal frequency is synonymous with a bigger difference between running speed and visual speed does not seem valid.

We acknowledge the reviewer's observation that our model does not explicitly establish a direct relationship between visual speed and running speed. In our study, we increased the speed of visual flow to explore its impact on dVf and hVf neuron responses. We aimed to determine if a preference for slower or faster visual flow speed contributed to the observed clustering within excitatory L2/3 neurons.

Furthermore, we draw parallels with previous research (Zmarz & Keller, 2016) on mismatch neurons, where increased activity was observed with higher visual flow prediction mismatches in open-loop scenarios.

Moreover, other studies suggest that **a preference for static or slower moving gratings explains the increases in firing rate observed in response to the visual flow perturbation, irrespective of predictions based on running direction (Muzzu, 2021).**

In our model, by varying visual flow speed as the sole parameter, we specifically targeted the microcircuit responsible for visual flow integration, while keeping locomotion integration pathways constant. This allowed us to investigate the effects of visual flow speed on neural responses.

In this sense, we wanted to make it clear that we are not establishing a direct link between our dVf and hVf neuron and experimental mismatch neurons, so we rephrased the manuscript as follows:

“Considering the sensitivity of dVf and hVf neurons to drifting gratings, it was plausible to hypothesize that hVf neurons might exhibit a preference for static gratings over drifting ones, and vice versa for dVf neurons, aligning with experimental observations where visual flow and running speed were uncoupled (Muzzu, 2021).
To further investigate this, the grating drift speed was increased to 8 Hz, leading to a reclassification of excitatory L2/3 neurons based on their response to this new stimulus. Interestingly, our findings revealed that neuron assignments to dVf and hVf classes remained largely unaffected, with the presynaptic current responses maintaining a comparable magnitude (Fig 6B-C). This outcome stands in contrast to the temporal frequency preferences exhibited by perturbation-sensitive neurons (Muzzu, 2021), as well as

the behavior of mismatch-responsive neurons, which demonstrated linear scaling of responses with the difference between running speed and visual flow speed (Zmarz, 2016; Attinger, 2017).

This observation highlights a specific limitation within the Billeh model when handling different temporal dynamics of visual stimuli."

Reference:

Zmarz, P., & Keller, G. B. (2016). Mismatch receptive fields in mouse visual cortex. Neuron, 92(4), 766-772.

2.13. *Line 480: "These consistent responses may be caused by the inherent preferences of the neurons. 480 Particularly, it is plausible that hVf neurons lean towards slower visual flow speeds, 481 such as static gratings, while dVf neurons might lean towards faster visual flow speeds, 482 implying a positive response to increased speed. To investigate this, the responses of 483 these neurons were examined across various visual flow temporal frequencies (1: 9: 1 484Hz) while maintaining a vertical grating orientation. The preferred frequency for each 485neuron in this orientation was then identified as the frequency at which the dVf/hVf 486neuron received the highest/lowest input current during visual flow. Remarkably, both 487dVf and hVf neurons displayed similar distributions of preferred frequencies (Fig. S7B), 488 peaking around 5 Hz."*

Clarification – is this only about non-direction selective cells discussed in the previous paragraph?

The paragraph in question exclusively pertains to perturbation-sensitive neurons. To clarify, we have reworded it as follows:

"The consistent responses of perturbation-sensitive neurons may be stem from their intrinsic preferences. Specifically, it is plausible that hVf neurons tend to favor slower visual flow speeds, such as static gratings, while dVf neurons lean towards faster visual flow speeds, indicating a positive response to increased speed. To investigate this, we examined the responses of these neurons across various visual flow temporal frequencies ($1:9:1$ Hz) while maintaining a vertical grating orientation. The preferred frequency for each neuron in this orientation was determined as the frequency at which the dVf/hVf neuron received the highest/lowest input current during visual flow. Remarkably, both dVf and hVf neurons exhibited similar distributions of preferred frequencies Fig S8B), peaking around 5 Hz."

2.14. *Line 493: "Finally, to further test whether perturbation-sensitive neurons encoded a sudden 493change in visual flow, we presented the model with black-to-white full-field flash and 494 tracked the responses of excitatory L2/3 neurons (Fig. 7)."*

Terming a flash a change in visual 'flow' is a bit misleading. It's a change in visual 'input' but there's no 'motion' or 'flow' here, to my understanding.

We agree with your point, it is more accurate to say that perturbation-sensitive neurons encoded a change in visual input rather than in visual flow, and we have revised the text accordingly. The updated passage now reads:

"To conclusively determine if perturbation-sensitive neurons are indeed encoding visual perturbations, i.e. sudden changes in visual input, we presented the model with a black-to-white full-field flash and tracked the responses of excitatory L2/3 neurons (Fig. 7 and Fig. S9)."

2.15. *Fig 6c: the stimulus panels are hard to see properly and would benefit from being enlarged.*

We appreciate the suggestion and have enlarged the stimulus panels in Fig 6c to improve visibility. Additionally, we have adjusted the y-axis scales to enhance the distinction in temporal frequency.

2.16. *Line 571: "indicative of their role in detecting 571 general visual flow perturbations. This was confirmed through a full field flash visual 572 stimulus (Fig. 7)."*

As mentioned previously, I think 'general changes in visual input' or similar would be more appropriate language.

We have now modified the text as suggested, replacing 'general visual flow perturbation' with 'general visual input perturbations' in line 571.

2.17. *Line 585: "Recent 585 experimental evidence shows that mouse visual areas prioritize the encoding of 586 potentially fast-changing and behaviorally relevant visual features [44]."*

I'm not sure this work 'shows' this, but more provides evidence towards it. 'suggests', or 'indicates' may be more appropriate than 'shows'.

We have revised the sentence accordingly. It now reads: "Recent experimental evidence suggests that mouse visual areas prioritize the encoding of potentially fast-changing and behaviorally relevant visual features [44]."

2.18. *Paragraph starting line 575: "It is important to acknowledge the role of additional factors, such as 575 locomotion-related inputs [15, 17–20, 52] and feedback from higher visual areas [57], in 576 modulating V1 neuron responses to visual flow*

perturbations…"

For this section, it would be useful to explain how your results fit into this research. For example, can you state anything about how your results clarify the role of these top-down inputs in predictive coding?

While our findings demonstrate the intriguing emergence of e23 neuron groups solely with visual input, establishing a direct link between these neurons and predictive error processing requires the inclusion of top-down inputs, which we acknowledge as a critical aspect. We will incorporate this perspective into our model and investigate it further as part of our ongoing research.

We sincerely hope that our responses and the corresponding revisions adequately address the concerns and suggestions raised by both reviewers. We believe that these changes have significantly improved the quality and clarity of our manuscript, making it a more valuable contribution for publication in PLOS Computational Biology.

Overall, we appreciate the constructive feedback and believe that the results reported in our manuscript offer important insights into the mechanisms underlying visual responses and therefore should be of broad interest to the scientific community.

Sincerely,

Claudio Mirasso (on behalf of all authors).
