# Supplementary material for: Modeling circuit mechanisms of opposing cortical responses to visual flow perturbations
Source: PLoS Comput Biol. 2024 Mar 7;20(3):e1011921. doi: 10.1371/journal.pcbi.1011921 (PMC10950248; doi:10.1371/journal.pcbi.1011921)
Supplement: Attachment — Submitted filename: Fraileetal2023-ModelOfPredictiveCoding.docx [file pcbi.1011921.s015.docx]

**Summary of work**

This is an interesting and useful study investigating if the combination of feedforward inputs and recurrent V1 circuit mechanisms can cause produce both hyperpolarising and depolarising responses to the sudden onset of movement of drifting gratings stimuli. The authors frame the work as relevant to studies of predictive coding in mouse V1, which have used similar stimulus protocols.
To achieve this, the authors perform simulations using an existing biologically realistic computational model of mouse V1 which receives feedforward inputs from LGN.

The authors first describe how neurons specifically in L2/3 can be split evenly into having depolarising (dVf) and hyperpolarising (hVf) responses to stimulus movement onset. They show that the baseline properties of these neurons differ and that their responses are in antiphase. They then explore the anatomical and functional connectivity of dVf and hVf neurons to show that these opposing responses arise from different functional connectivity from LGN inputs and recurrent circuits. Finally, the authors investigate to what extent direction selectivity of neurons can explain dVf and hVf responses and find a subset of ‘perturbation’-selective neurons that respond to movement onset irrespective of the direction of stimulus movement.

Overall, the manuscript adds valuable circuit-level mechanistic insight into how putative ‘prediction error’ responses can arise in mouse V1 in the absence of explicit top-down input (e.g. from locomotion-related signals).

**Comments**

1. My main comments are regarding how parts of the manuscript are ‘framed’, or other language use.

   The stimulus paradigm itself is not really testing predictive coding explicitly – the stimulus is stationary, moves, and then stops moving. There is no explicit prediction here about what the stimulus is likely to do, and indeed behaviour-related variables are not included in the model. Instead, the paradigm reflects stimulus protocols previously used in studies of predictive coding (e.g. [12] and [16]) and seeks to understand if and how bidirectional responses can arise in the absence of sensorimotor expectation.

   The authors state it well in the discussion (line 515)
   *This study aimed to explore the emergence of distinct perturbation-responsive V1 neuron classes in the absence of a sensorimotor expectation using the Billeh model, a biologically realistic computational model [32].*

   I felt that a statement such as this could come much earlier in the introduction. Based on the current introduction which, I found myself expecting simulations that more explicitly test predictive coding of visual flow (i.e. using sensorimotor expectation). Given that the current study is addressing a very specific question in the field, I suggest making this objective clearer at an early point in the manuscript, to set appropriate expectations for the reader.
2. Line 2:   *The brain’s ability to perceive and respond to rapidly changing visual stimuli is a fundamental aspect of sensory processing, vital for the survival and adaptive behavior of* animals in their natural environments [1].
   The reference here (a motion coherence paradigm in mice with recordings in V1) does not seem appropriate to the statement made.
3. At time the introduction felt longer than it needed to be, and at times unfocused e.g. Line 8

*Understanding the neural basis of motion perception and the response to visual flow perturbations represents a critical goal in sensory neuroscience, as it would shed light on the underlying processes of attentional modulation and decision-making [5–7].*

I don’t particularly disagree with the statement, but it doesn’t seem overly necessary to this specific study.

4. Line 12*: The precise timing with which the brain perceives unexpected stimuli*

   To say the ‘brain perceives’ seems strange. Perhaps ‘signals’ is more appropriate?
5. Line 41 and elsewhere: *This segregation is indicative of the neurons’ response to visual flow perturbations*

   I appreciate ‘visual flow perturbations’ is language previously used in other studies, but I would find the text clearer if it be more explicit, e.g. ‘stimulus movement onset/halt’
6. Line 318*: cortical microcircuits have shown that PE neurons may require a balance between*

Would be preferable not to use the acronym PE for ease of reading

7. *Line 325: In fact, many hVf neurons lack direct connections to the LGN, indicated by zero in-degree values (Fig. 4A).*

It would be useful to briefly define ‘in-degree’ explicitly here.

8. Line 329: *The analysis of the connections of excitatory L2/3 neurons revealed a pronounced 329 tendency for neurons of the same class to exhibit a higher probability (Fig. 4B ) and 330*

*strength (Fig. 4C ) of connections.*

Is this really a pronounced tendency? The differences in probability of connection seem quite small (Fig. 4B). It would be useful to state the % or fractional difference (i.e. 10% more likely) and test the statistical significance. The strength of synaptic weights seems more 'pronounced' – it would be nice to have these differences tested statistically.

9. Line 332: *This pattern indicates a rich club structure in dVf and hVf neurons*

   It would be helpful to define ‘rich club’ structure rather than relying on a later example as a definition.
10. Line 344: *However, dVf 345 neurons possess slightly stronger incoming recurrent synapses, primarily due to 346 contributions from excitatory L4 neurons (Fig. 4E ) and increased LGN input.*

    What is exactly is meant here? Does it mean bottom-up LGN inputs AND recurrent cortical connections. I was confused by this statement. Perhaps a comma is missing after ‘(Fig 4E)’?
11. Figure 5: These are interesting findings, but I struggled to parse the figures properly. E.g. Figuring out which boxplots belong to which x-axis label is difficult. The two different colours used are also hard to parse for panels this small. Additionally, the different y-axis scales limited comparisons of all the different cell types. Perhaps the data could be replotted in a clearer way, potentially by removing weights that are not significantly different from 0 across all the panels (which could be included as a supplementary figure instead). It would also be beneficial to show which differences between hvf and dvf neurons are statistically significant.
12. *Line 431: To further investigate this, the grating drift speed was increased to 8 Hz, leading to a 431reclassification of excitatory L2/3 neurons based on their response to this new stimulus. 432 Interestingly, we found that neuron assignments to dVf and hVf classes were not 433 significantly affected (Fig. 6B and C ), contrary to what was found in mismatch 434 responsive neurons, where their responses scaled linearly with the difference between 435 running speed and visual flow speed [11]. This observation underscores a specific 436 limitation in the Billeh model when processing different temporal dynamics of visual 437 stimuli.*

    Given that no relationship between visual speed and running speed is established in the simulation (indeed, as far as I can tell, behaviour is not explicitly modelled in any way), to imply that higher temporal frequency is synonymous with a bigger difference between running speed and visual speed does not seem valid.
13. Line 480: *These consistent responses may be caused by the inherent preferences of the neurons. 480 Particularly, it is plausible that hVf neurons lean towards slower visual flow speeds, 481 such as static gratings, while dVf neurons might lean towards faster visual flow speeds, 482 implying a positive response to increased speed. To investigate this, the responses of 483 these neurons were examined across various visual flow temporal frequencies (1 : 9 : 1 484Hz) while maintaining a vertical grating orientation. The preferred frequency for each 485neuron in this orientation was then identified as the frequency at which the dVf/hVf 486neuron received the highest/lowest input current during visual flow. Remarkably, both 487dVf and hVf neurons displayed similar distributions of preferred frequencies (Fig. S7B ), 488 peaking around 5 Hz.*

Clarification – is this *only* about non-direction selective cells discussed in the previous paragraph?

14. Line 493: *Finally, to further test whether perturbation-sensitive neurons encoded a sudden 493change in visual flow, we presented the model with black-to-white full-field flash and 494 tracked the responses of excitatory L2/3 neurons (Fig. 7).*

    Terming a flash a change in visual 'flow' is a bit misleading. It's a change in visual 'input' but there's no 'motion' or 'flow' here, to my understanding.
15. Fig 6c: the stimulus panels are hard to see properly and would benefit from being enlarged.
16. Line 571: *, indicative of their role in detecting 571 general visual flow perturbations*. *This was confirmed through a full field flash visual 572 stimulus (Fig. 7).*

    As mentioned previously, I think 'general changes in visual input' or similar would be more appropriate language.
17. Line 585: *Recent 585 experimental evidence shows that mouse visual areas prioritize the encoding of 586 potentially fast-changing and behaviorally relevant visual features [44].*

    I'm not sure this work 'shows' this, but more provides evidence towards it. 'suggests', or 'indicates' may be more appropriate than ‘shows’.
18. Paragraph starting line 575*: It is important to acknowledge the role of additional factors, such as 575 locomotion-related inputs [15, 17–20, 52] and feedback from higher visual areas [57], in 576 modulating V1 neuron responses to visual flow perturbations…*

    For this section, it would be useful to explain how your results fit into this research. For example, can you state anything about how your results clarify the role of these top-down inputs in predictive coding?
